# Root form and canal anatomy of maxillary first premolars: a cone-beam computed tomography study

**DOI:** 10.1007/s10266-021-00670-9

**Published:** 2021-10-29

**Authors:** Katarzyna Olczak, Halina Pawlicka, Witold Szymański

**Affiliations:** 1 Department of Endodontics, Medical University of Lodz, Pomorska 251, 92-213 Lodz, Poland; 2 Institute of Materials Science and Engineering, Lodz University of Technology, Stefanowskiego 1/15, 90-924 Lodz, Poland

**Keywords:** Canal configuration, Cone-beam computed tomography, Endodontics, Maxillary premolars, Root morphology

## Abstract

The aim of this study was to evaluate the root and canal morphology of permanent maxillary first premolars in a Polish population using cone-beam computed tomography scanning (CBCT) and to compare the classifications by Vertucci and Ahmed et al. Images of 350 maxillary first premolars were analyzed. Scans were obtained from 226 patients: 131 women and 95 men. The root canal configurations were classified according to Vertucci and a new system by Ahmed et al. In addition, the number of roots and the level where roots bifurcated were identified. The results were submitted to statistical analysis. Most maxillary first premolars had two roots (69.1%). Most bifurcations were located in the coronal part of the root (44.2%) and the least in the apical part (15.3%). Bifurcation in the coronal part of the root was observed more often in the teeth of men than women. In turn, bifurcation in the central or apical part was significantly more common in women than in men. The most common canal configuration of the maxillary first premolars was type IV (78.2%) according to Vertucci and ^2^FPB^1^P^1^ (65.4%) according to the new classification. Among the remaining cases, almost all types of canals described by Vertucci, and many combinations of codes given in the new classification were demonstrated. The maxillary first premolars displayed a wide range of root and canal anatomical variations. The new system for classifying canal morphology based on Ahmed et al. is more accurate than the Vertucci classification.

## Introduction

**Fig. 1:**
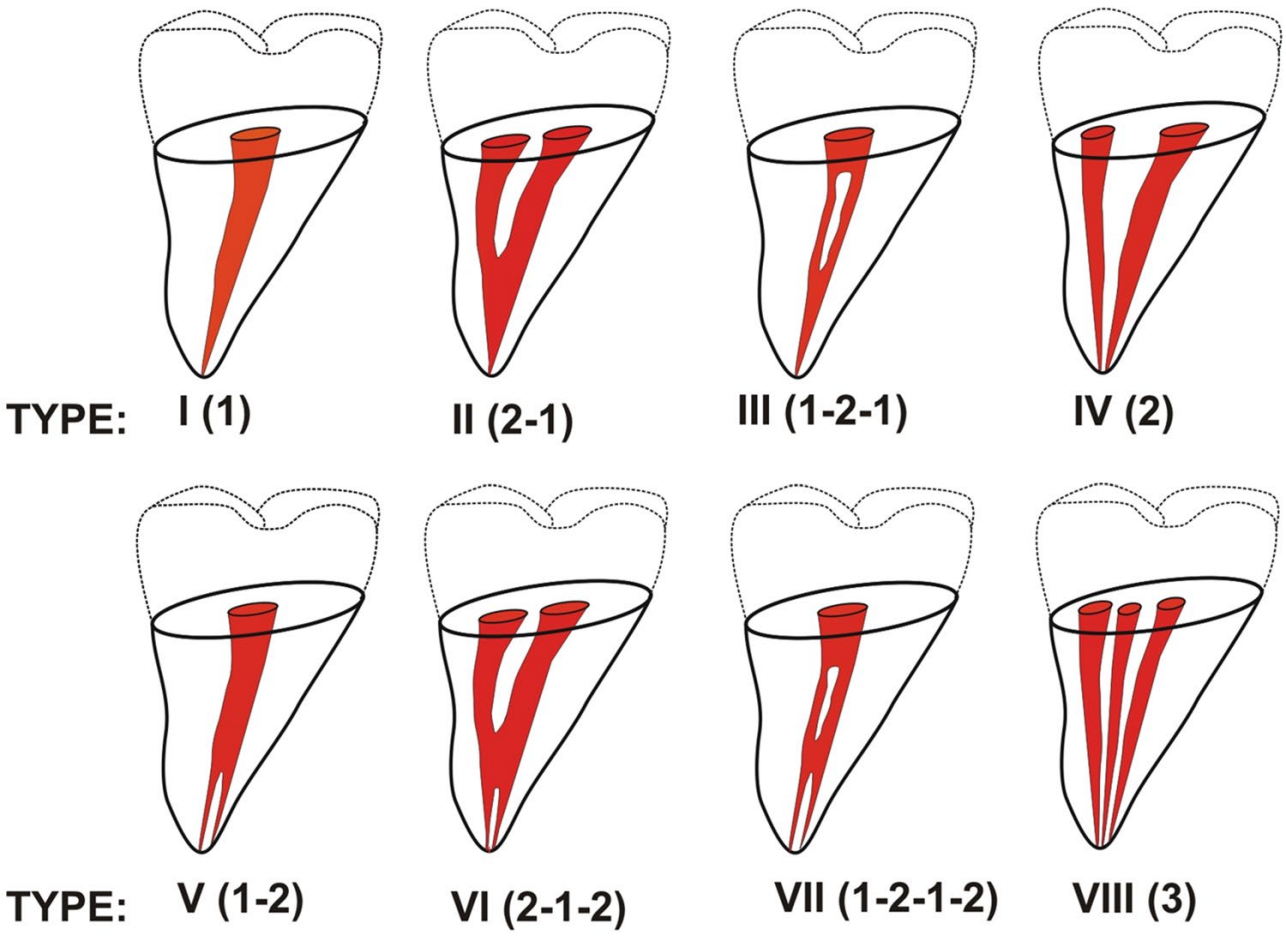
Diagrammatic representation of Vertucci’s canal configuration

The success of endodontic treatment is greatly affected by the location of the root canals. An awareness of the complex anatomy of root canal system and the possible occurrence of anomalies in the external and internal structure is very useful during the trepanation, preparation, and filling of the root canals [1]. In clinical work, the main tools used to assess the root canal anatomy are the dental operating microscope and radiological imaging [1, 2]. Although X-rays are usually taken before and after root canal treatment, difficult diagnostic and therapeutic merit the use of cone-beam computed tomography (CBCT) [3]. Volumetric imaging allows the dentist to accurately trace the morphology of the canals and roots and see anatomical details that are invisible or hardly noticeable on X-rays. Using tomography, clinicians are able to analyze not only the number but also the configuration of root canals [4]. This is especially useful when treating teeth with varied anatomy which are difficult to prepare. Such teeth include, among others, maxillary first premolars [5, 6]. Although they are usually dual rooted, they have very diverse morphology of root canals. All types of canals given in the Vertucci classification (1984) have been found to occur in this group of teeth [6–8]. The Vertucci classification (Fig. 1) is very often used in scientific investigations, teaching, and clinical work. It is also more accurate than the Wein classification (1969, first root canal classification), which only contains four types of canals, which is twice as small as the Vertucci classification [8, 9]. According to Wein’s classification distinguishes the following root canal types:

- Type I (1-1): Single canal running from orifice to apex.
- Type II (2-1): Two canals arising from the pulp chamber and uniting into a single one during its course.
- Type III (2-2): Two canals running separately from orifice to apex.
- Type IV (1-2): One canal arising from the floor of the pulp chamber and dividing into two during its course [9].

However, the anatomy of the teeth is so diverse that both Wein’s classification and Vertucci’s classification have become insufficient to describe the newly discovered root canal morphology. Therefore, with the development of new diagnostic methods, and the possibilities for examining the structure of dentition, more modern classifications have appeared. In 2001, Gulabivala et al. [10], following a study of mandibular molars in a Burmese population, added seven new configurations to Vertucci’s classification: type I (3-1), type II (3-2), type III (2-3), type IV (2-1-2-1), type V (4-2), type VI (4-4), and type VII (5-4).

Also, in 2004, based on a study of root canal configuration in 2800 maxillary and mandibular permanent teeth among a Turkish population using a clearing technique, Sert and Bayirli [11], added 15 new configurations: type IX (1-3), type X (1-2-3-2), type XI (1-2-3-4), type XII (2-3-1), type XIII (1-2-1-3), type XIV (4-2), type XV (3-2), type XVI (2-3), type XVII (1-3-1), type XVIII (3-1), type XIX (2-1-2-1), type XX (4-4), type XXI (4-1), type XXII (5-4), and type XXIII (3-4).

In 2009, Al-Qudah [12], presented four new types of root canal configurations of mandibular molars in a Jordanian population: type XX (2-3-1), type XXI (2-3-2), type XXII (3-2-1), and type XXIII (3-2-3).

Although the new classifications include more root canal types, all have two basic limitations:

1. All contain a strictly defined number and configuration of root canals; as such, if a new root canal configuration is discovered, it must be named as “unclassified” and/or a new type must be created. In studies by Filpo-Perez [13] and Karobari [14], 13% and 3% of samples, respectively, did not fit in Vertucci’s classification or its supplements. Additionally, the same root canal configuration can be assigned different classifications, e.g., the 4-4 configuration corresponded to Type VI by Gulabivala [10] and Type XX by Sert [11].
2. The classifications refer only to the configuration of the canals and do not take into account the morphology of the roots or the obvious “connection” of the canals with the roots.

**Fig. 2:**
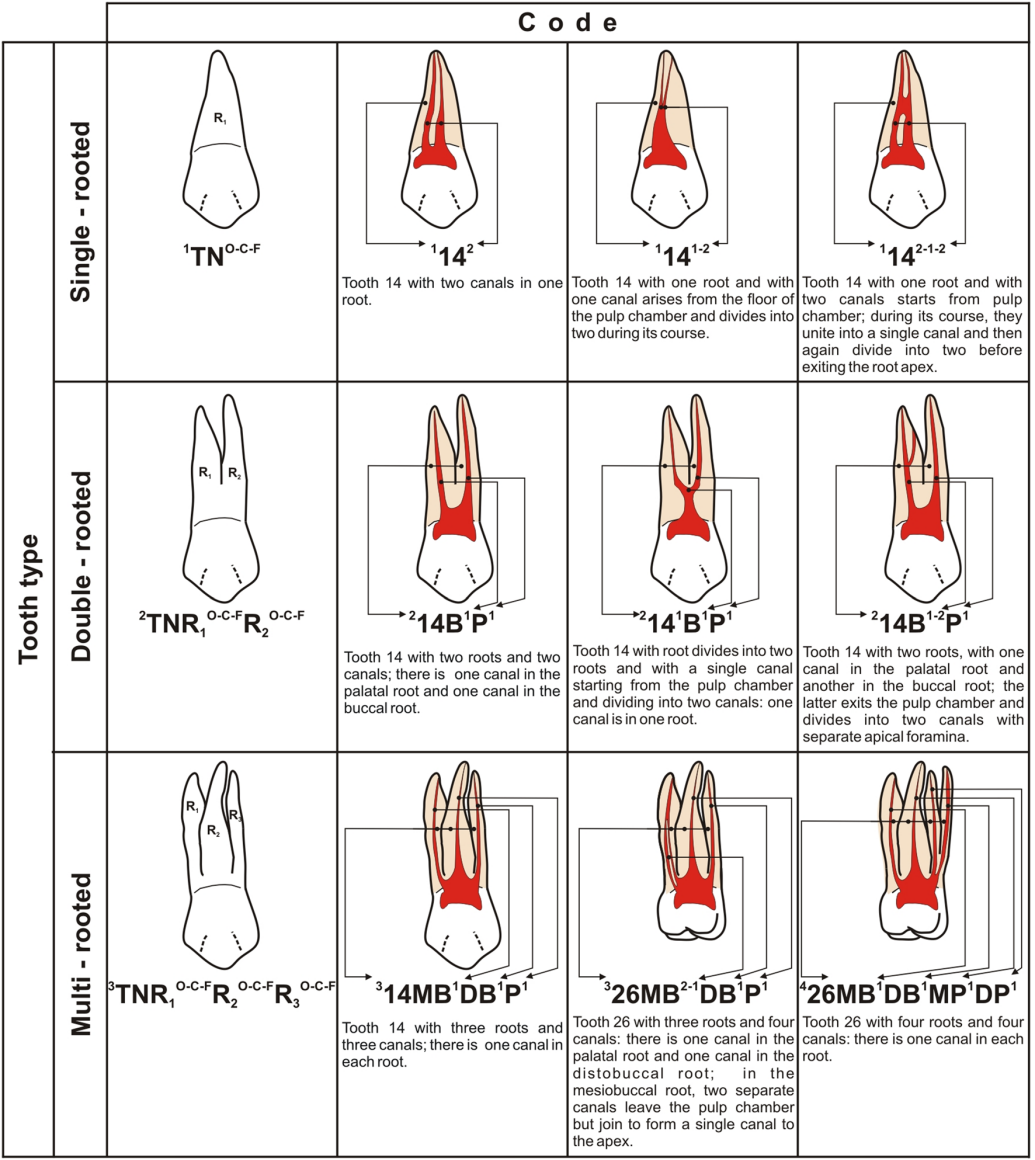
Diagrammatic representations of the new system for classifying root and canal morphology according to Ahmed et al*.*’s classification (Ahmed et al*.* [15]). *TN* Tooth Number. Superscript on the left of TN: Number of roots, Superscript on the right of TN: In single-rooted tooth: root canal configuration type. In double-rooted tooth: Root canal configuration type on the right of each root. O-C-F = root canal configuration type starting from Orifice passing through Canal to Foramen. *O* orifice, *C* canal, *F* Foramen, *R* root, *B* Buccal, *P* Palatal, *DB* Distobuccal, *MB* Mesiobuccal, *DP* Distopalatal

Due to some imperfections of the existing classifications, a new classification was created in 2017 by Ahmed et al. [15]. This classification has no strictly defined number of canal types. Instead, the system is based on “codes” assigned individually to a given canal/root structure, and can hence account for even the most complex root canal configuration (Fig. 2). Additionally, one record/code contains information on both the number and the course of canals, as well as the number and structure of roots [15, 16].

The Vertucci classification (and others similar) contains some inaccuracies and does not provide information on the canal configuration in relation to the number and shape of roots [15, 16]. In contrast, Ahmed et al. [15] provide a better indication of the anatomy of the teeth. For example, a type IV canal according to Vertucci can denote a tooth with two canals in one root, as well as a tooth with two canals in two separate roots (Fig. 2). According to Ahmed et al. [15], the presence of two root canals in one root, e.g., for tooth 14, is indicated by ^1^14^2^, while two canals in two different roots, e.g., for tooth 14, are indicated by ^2^14B^1^P^1^ (an explanation of the entries/codes is given in Fig. 2). The same is true, for example, with type V or VIII (Vertucci classification). The “type V” configuration gives is no information whether a division of the root exists, i.e., whether in the periapical area, two canals are in one common root or there are (after separation) two single canals in two separate roots; in contrast, according to the new Ahmed et al.’s classification, ^1^14^1–2^ indicates a common root, whereas ^2^14^1^B^1^P^1^ indicates a split canal and a split root (Fig. 2). The situation with Vertucci type VIII is even more complicated, as this can indicate three canals in three separate roots, or in two roots or even only in a single root. According to the Ahmed classification, each of these cases has a separate code and gives information about both the number of roots and the number and “course” of canals in each of the roots: three root canals in three separate roots in tooth 14 are given as ^3^14MB^1^DB^1^P^1^; the presence of two roots, in which the canal is divided into two in one of them, such as in the buccal canal (resulting in three canals in two roots), is given by ^2^14B^1−2^ P^1^ (Fig. 2). Thus, the Ahmed et al.’s [15] classification allows the anatomy of the teeth to be to described in one code (in one record), even in the case of multi-rooted teeth, and gives simple, clear, and complete information about the configuration of the canal/canals in each root, not just about the number of canals in the root (Fig. 2).

Additionally, while classifications exist for both roots and canals, they do not apply to all groups of teeth and they do not accurately reflect the “course” of the canal in the root nor whether, for example, two canals begin with two separate paraventricular orifices or the canal is divided two only at the tip of the root. For example, Zhang et al. [17], offer the following classification for mandibular molars only, according to the number of roots and the number of canals in each root:

Variant 1: Two separate roots, a mesial and a distal root, with one canal in each root.

Variant 2: Two separate roots, a mesial and a distal root, with one canal in the mesial root and two canals in the distal root.

Variant 3: Two separate roots, a mesial and a distal root, with two canals in the mesial root and one canal in the distal root.

Variant 4: Two separate roots, a mesial and a distal root, with two canals in each root.

Variant 5: Three separate roots, mesial, distobuccal, and distolingual, with one canal in each root.

Variant 6: Three separate roots, with two canals in the mesial root and one canal each in the distobuccal and distolingual roots.

Variant 7: Four separate roots, mesiobuccal, mesiolingual, distobuccal, and distolingual, with one canal in each root.

The above classification applies only to mandibular molars and only to the number of roots and root canals.

The Ahmed et al.’s [15] classification thus provides a more precise description of tooth morphology (in all teeth) than the classifications used so far. However, the Vertucci classification is one of the best-known classifications in dentistry, and the most widely used systems for recording the internal anatomy of teeth in research papers. The Vertucci classification has played a very important role in the development of research on tooth anatomy.

The purpose of this study was to evaluate the root and canal morphology of permanent maxillary first premolars in a Polish population using cone-beam computed tomography scanning (CBCT), and to compare the findings based on two specific classification methods (Vertucci/Ahmed).

## Materials and methods

All experimental procedures in this study were approved by the Ethics Committee of the Medical University of Lodz (Protocol no. RNN/166/15/KE). Cone-beam computed tomography (CBCT) scans of the maxilla of 226 Polish patients (131 women and 95 men, average age 42.5 years) were examined. All were taken from May 2015 to December 2018 in the Dental Hospital of the Medical University of Lodz for diagnosis or planning of dental treatment. Images of 350 maxillary first premolars were analyzed.

All images were taken using a Gendex GXDP-800^®^machine (Gendex^®^) with image capture parameters set at 90 kV and 8.0 mA, and an exposure time of 2.3 s.

The inclusion criteria were as follows:

- Each patient had to have at least one (or two) maxillary first premolar.
- Maxillary premolars were free of root canal treatment.
- Premolars could not have reconstructions such as post-reconstruction that would make it difficult to assess their anatomy.
- The teeth had to be completely shaped with fully developed apices.
- The teeth had not undergone resorption.

**Fig. 3:**
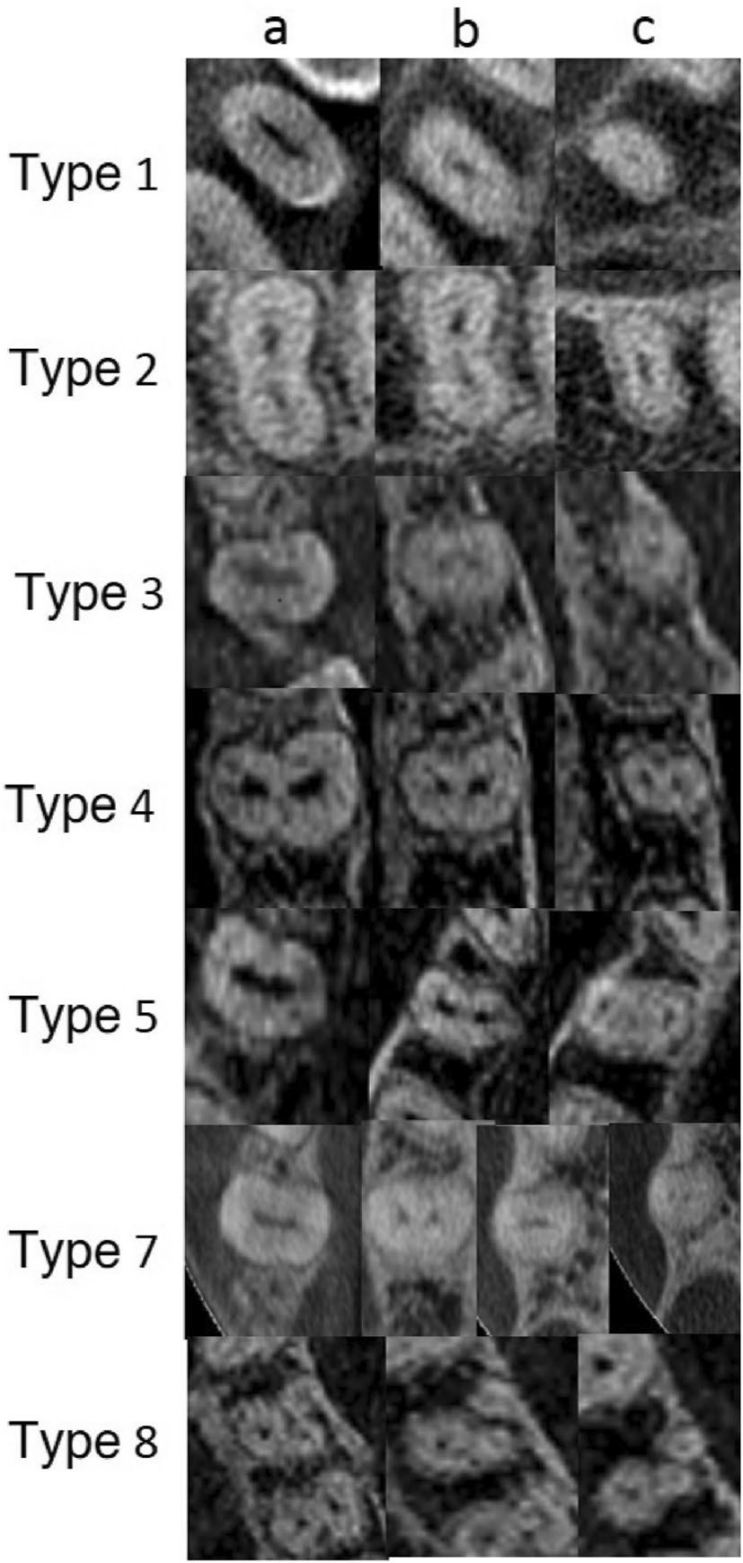
CBCT scanning in the horizontal plane of the coronal (**a**), middle (**b**), and apical thirds (**c**) of the root demonstrated variations in canal morphology according to Vertucci classification

The scans were analyzed using In Vivo 5 software, version 5.3 “Anatomage” (Fig. 3). All scans were evaluated separately by two endodontists and any disagreement was discussed until a consensus was reached. The CBCT images were analyzed as follows: axial, coronal, and sagittal two-dimensional sectional images were displayed. The anatomy of the teeth was assessed according to the following parameters:

1. Number of roots

The number of roots was determined as follows:

A single-rooted tooth—a tooth with a clear single root.

A double-rooted tooth—a tooth with bifurcated roots (regardless of partial or complete root separation).

A three-rooted tooth—a tooth with three roots (regardless of partial or complete separation).

2. Root bifurcation

In addition, the level of root bifurcation was identified. Using a software ruler, each root was divided into three thirds:

1- Coronal section—from the cemento-enamel junction to 1/3 of the root length.

2- Middle section—from 1/3 to 2/3 of the root length.

3- Apical section—from 2/3 of the root length to the radiographic apex.

3. Root canal configuration

The root canal configurations were classified according to Vertucci [6] and the new system by Ahmed et al*. *[15]

The results were submitted to statistical analysis using the Chi-square test with Yates’ correction (*p* < 0.05).

## Results

### Root morphology

Of all the examined teeth, two-rooted forms were most common (69.1%). Single-rooted teeth were recorded much less frequently (28.3%). Three roots in the maxillary first premolars were found in only a few cases (2.6%) (Table 1). The percentages of teeth with particular numbers of roots were quite similar between men and women. (Table 1). No statistically significant difference (*p* > 0.05) was found in the prevalence of root numbers between teeth 14 and 24 (Table 1).

**Table 1:** Frequency distribution of number of roots (% of teeth) in maxillary first premolars (MFP) according to gender and tooth position

MFP	One root (%)	Two roots (%)	Three roots (%)	Total (%)
Gender				
Male	40 (27.6)	99 (68.3)	6 (4.1)	145 (100.0)
Female	59 (28.8)	143 (69.7)	3 (1.5)	205 (100.0)
Total	99 (28.3)	242 (69.1)	9 (2.6)	350 (100.0)
Tooth position				
Right (14)	42 (23.5)	132 (73.7)	5 (2.8)	179 (100.0)
Left (24)	57 (33.3)	110 (64.4)	4 (2.3)	171 (100.0)
Total	99 (28.3)	242 (69.1)	9 (2.6)	350 (100.0)

Gender: chi^2^ = 2.432; *p* = 0.296

Tooth position: chi^2^ = 4.203; *p* = 0.122

Bifurcation occurred most often in the coronal third of the root (44.2%), followed by the middle third (40.5%), and occurred least often in the apical third (15.3%) (Table 2).

**Table 2:** Levels of root bifurcations (in teeth with two roots) in maxillary first premolars (MFP) according to gender and tooth position, (% of teeth)

MFP	Coronal part (%)	Middle part (%)	Apical part (%)	Total (%)
Gender				
Male	58 (58.6)	31 (31.3)	10 (10.1)	99 (100.0)
Female	49 (34.3)	67 (46.8)	27 (18.9)	143 (100.0)
Total	107 (44.2)	98 (40.5)	37 (15.3)	242 (100.0)
Tooth position				
Right (14)	55 (41.7)	54 (40.9)	23 (17.4)	132 (100.0)
Left (24)	52 (47.3)	44 (40.0)	14 (12.7)	110 (100.0)
Total	107 (44.2)	98 (40.5)	37 (15.3)	242 (100.0)

Gender: chi^2^ = 14.264; *p* = 0.0008

Tooth position: chi^2^ = 1.304; *p* = 0.521

A statistically significant difference between sexes was observed regarding the prevalence of particular root split levels (p < 0.05). Bifurcation in the coronal third of the root was observed more often among men than women (58.6 vs. 34.3%, *p* < 0.05); however, bifurcations in the middle or apical sections were significantly more common in women than men (middle third 46.8 vs. 31.3%; apical third 18.9 vs. 10.1%, *p* < 0.05) (Table 2).

No significant difference was observed between teeth 14 and 24 regarding the prevalence of particular root split levels (*p* > 0.05) (Table 2).

### Root canal morphology

Analysis of root canal systems according to the Vertucci classification:

Among all teeth, the most common type of root canal according to the Vertucci classification was type IV (78.5%), followed by type II (8.6%), type V (5.1%), type VIII (2.9%), type III (2.6%), and type I (1.7%). Type VII was found only in the case of two teeth (0.6%), and type VI was not observed at all (Table 3).

**Table 3:** Prevalence of root canal types (Vertucci classification) in maxillary first premolars (MFP) according to gender and tooth position

MFP	Types of canal configuration								
	I (%)	II (%)	III (%)	IV (%)	V (%)	VI (%)	VII (%)	VIII (%)	Total (%)
Gender									
Male	2 (1.4)	9 (6.2)	2 (1.4)	119 (82.1)	6 (4.1)	0 (0.0)	0 (0.0)	7 (4.8)	145 (100.0)
Female	4 (2.0)	21 (10.2)	7 (3.4)	156 (76.1)	12 (5.8)	0 (0.0)	2 (1.0)	3 (1.5)	205 (100.0)
Total	6 (1.7)	30 (8.6)	9 (2.6)	275 (78.5)	18 (5.1)	0 (0.0)	2 (0.6)	10 (2.9)	350 (100.0)
Tooth position									
Right(14)	3 (1.7)	13 (7.3)	4 (2.2)	144 (80.4)	8 (4.5)	0 (0.0)	1 (0.6)	6 (3.3)	179 (100.0)
Left(24)	3 (1.8)	17 (9.9)	5 (2.9)	131 (76.7)	10 (5.8)	0 (0.0)	1 (0.6)	4 (2.3)	171 (100.0)
Total	6 (1.7)	30 (8.6)	9 (2.6)	275 (78.5)	18 (5.1)	0 (0.0)	2 (0.6)	10 (2.9)	350 (100.0)

Gender: chi^2^ = 4.905; *p* = 0.556

Tooth position: chi^2^ = 1.327; *p* = 0.970

The prevalence of particular Vertucci root canal types did not differ significantly according to sex (*p* > 0.05) (Table 3). No statistically significant difference (*p* > 0.05) was found in the prevalence of particular types of canals between teeth 14 and 24 (Table 3).

Analysis of root canal systems according to the new classification (Ahmed et al*.*) [15]:

The analysis of root canal systems according to the new classification showed that the most common variant observed among all first upper premolars was code ^2^FPB^1^P^1^ (65.4%). This was followed by code ^1^FP^2^ (13.1%), code ^1^FP^2−1^ (8.6%), code ^2^FP^1^B^1^P^1^ (3.4%), code ^1^FP^1−2–1^, and ^3^FPMB^1^DB^1^P^1^ (2.6% each). Code records ^1^FP^1^ and ^1^FP^1−2^ were much less common (1.7% each). In turn, code ^1^FP^1−2–1−2^ was present only in two teeth (0.6%), and code ^2^FP B^1−2^ P^1^ in only one tooth (0.3%). No examples of code ^1^FP^2−1–2^ were identified, corresponding to Vertucci type VI (Table 4). No statistically significant difference was observed in the prevalence of particular types of canals according to sex (*p* > 0.05) (Table 4). No significant differences in the prevalence of particular codes/canal types according to Ahmed et al. [15] were observed between teeth 14 and 24 (*p* > 0.05) (Table 4).

**Table 4:** Prevalence of root canal types (Ahmed et al.’s classification) in maxillary second premolars (MFP) according to gender and tooth position

MFP	Types of canal configuration											
	^1^X^1^ (%)	^1^X^2−1^ (%)	^1^X^1−2–1^ (%)	^1^X^2^ (%)	^1^X^1−2^ (%)	^1^X^2−1–2^ (%)	^1^X^1−2–1−2^ (%)	^2^XB^1^P^1^ (%)	^2^X^1^B^1^P^1^ (%)	^2^XB^1−2^P^1^ (%)	^3^XMB^1^DB^1^P^1^ (%)	Total (%)
Gender												
Male	2 (1.4)	9 (6.2)	2 (1.4)	26 (17.9)	1 (0.7)	0 (0.0)	0 (0.0)	93 (64.2)	5 (3.4)	1 (0.7)	6 (4.1)	145 (100.0)
Female	4 (2.0)	21 (10.2)	7 (3.4)	20 (9.8)	5 (2.4)	0 (0.0)	2 (1.0)	136 (66.3)	7 (3.4)	0 (0.0)	3 (1.5)	205 (100.0)
Total	6 (1.7)	30 (8.6)	9 (2.6)	46 (13.1)	6 (1.7)	0 (0.0)	2 (0.6)	229 (65.4)	12 (3.4)	1 (0.3)	9 (2.6)	350 (100.0)
Tooth position												
Right(14)	3 (1.7)	13 (7.3)	4 (2.2)	19 (10.6)	2 (1.1)	0 (0.0)	1 (0.6)	125 (69.8)	6 (3.3)	1 (0.6)	5 (2.8)	179 (100.0)
Left(24)	3 (1.8)	17 (9.9)	5 (2.9)	27 (15.8)	4 (2.4)	0 (0.0)	1 (0.6)	104 (60.8)	6 (3.5)	0 (0.0)	4 (2.3)	171 (100.0)
Total	6 (1.7)	30 (8.6)	9 (2.6)	46 (13.1)	6 (1.7)	0 (0.0)	2 (0.6)	229 (65.4)	12 (3.4)	1 (0.3)	9 (2.6)	350 (100.0)

Gender: chi^2^ = 8.055; *p* = 0.529

Tooth position: chi^2^ = 3.665; *p* = 0.932

X = MFP = maxillary first premolar

## Discussion

As much as 70% of the maxillary first premolars in the Polish population have two roots. Similar percentages have been recorded in Kosovar (70.1%) [18], Ugandan (73.3%) [19], Saudi (71.7%) [20], and Turkish (61.3%) [21] populations. However, the percentage of double-rooted premolars of the maxilla was found to be higher in an Indian population (91.7%) [22]. In contrast, some studies report a similar distribution of single- and two-rooted teeth in Singaporian [23], Spanish [24], and Egyptian populations [16], with only a slight predominance of two-rooted teeth: single-rooted teeth 46–49% and two-rooted teeth about 51% [16, 23, 24]. In addition, single-rooted forms were found to be more common in Japan (Sri Lanken-53.7%, Japanase-76.6%) [25] and Chinese (66%) [26] populations. Maxillary first premolars with three roots are relatively rare. In the present research, three-rooted maxillary premolars were found in only 2.6% of the analyzed cases. Similarly, Abella et al. [24] found three roots to be present in 2.6% of 430 premolars on the basis of CBCT images, while Neelakantan et al*.* [22] identified 2.3% of 350 teeth. Very similar or even identical results were also obtained by Chaparro et al*.* [27] and Pecora et al. [28]. Studies showing a higher percentage of three-rooted teeth are rare: maxillary premolars with three roots do not constitute more than a few percent of the total number of samples [18]. It is more common to observe a very low prevalence of three-rooted teeth (about 0.4–1%) or their absence (0%) [6, 10, 12, 13, 16, 18].

As most of the maxillary premolars examined in the present study, and in many previous studies, have two roots, an important issue is the location of bifurcation, i.e., the place of root separation. In the examined Polish population, the roots most often separated within two-thirds of the coronal section of the canal. Similar results were presented in a study by Peiris et al*.* [25] who found bifurcation to occur most often in the coronal or middle third of the canal in a Japanese population. In contrast, in the population of Egyptian residents, furcation was found to be much more common in the middle part (78.9%) than the apical (17.5%) or the coronal sections of the canal (3.6%) [16].

Our findings emphasize that the formation of the human body and the teeth varies according to population type. However, the choice of sample size and method may also influence the obtained results.

A few publications on tooth anatomy have discussed the relationship between root structure and sex [24, 29–31]. No statistically significant difference in the number of roots in the teeth of women and men was found in the present study, which is consistent with the results obtained from Spanish [24], Turkish [29], and Nepalese [30] populations. However, in a Chinese population, double-rooted teeth were twice as common in men (62.68%) than women (33.33%), and three-rooted teeth were observed in 3.73% of teeth from men, but were absent from women (0%) [31].

In the present research, a relationship was observed between bifurcation level and sex: in men, bifurcation was more common in the coronal third of the root than in women, while in women, it was more common in the middle and apical third of the canal than in men. This can be important information for the endodontist, when looking for the orifice of the second root canal. The root splitting site is the point where the next root canal (or canals) definitely begins, and a particular care should be taken at this stage of root canal instrumentation and later during canal filling.

Obviously, the canals can also separate and merge regardless of root division. A single canal in a single root, i.e., Vertucci type I code, is relatively rare, especially in lateral teeth. In the present study, the most common canal type in maxillary premolars was type IV according to the Vertucci classification. This is in line with the previous studies; for example, Type IV was observed in 78.4% and 76.9% in Turkish populations [29, 32], 74.7% in a Kuwaiti [33] population, and 79.7% in a Jordanian [7] population. In the cited studies, tooth morphology was determined using various techniques, such as clearing technique, X-rays, and tomography [7, 29, 32, 33].

A slightly higher prevalence of type IV (82.4%) was reported in the premolars of a Polish population based on an X-ray study of removed teeth by Lipski et al*.* [34] However, the study used fewer samples (142 teeth) than the present study and a different method to assess tooth anatomy.

Some studies indicate a lower prevalence of type IV canals, ranging from 50 to 69% depending on the study [20, 24, 25, 27, 31, 35]. In Chinese [36] and Indian [37] populations, type IV was found only to be present in 33–36% maxillary premolars.

In the studied Polish population, almost all types of root canals were observed in the remaining teeth. Only type VI was not found, which is consistent with the observations of many other authors [6, 22, 38–40]. Many reports indicate no examples of type III, VI, or type VII canals in the first premolar teeth [6, 7, 38–41]. In the present study, types III and VII were observed in only a few cases out of 350 teeth. Third canals (and type VIII) in maxillary first premolars are also rarely reported; and the maxillary premolars are most often regarded as two-canal teeth [6, 15, 16, 38–40]. Although type VIII is relatively rare in premolars, caution should be exercised whenever the tooth is suspected of having a third canal. A larger widening should be made to facilitate access to canals from the cheek side, since there are two buccal canals (proximal and distal) and one palatal canal. It is worth noting that while the Vertucci classification indicates the presence of three canals in type VIII, it does not indicate how exactly these canals run in the roots, i.e., how they divide, connect, or run as three independent canals from the coronal to periapical orifice, and whether they occur in one or two or three roots [15, 16].

Such knowledge is provided in the Ahmed et al*.*’s classification [15]. It should be emphasized that when three root canals were present, they most often appeared in three roots with a code record of ^3^FP MB^1^DB^1^P^1^. In one case, three canals were observed in two roots. This configuration is described by code ^2^FPB^1−2^ P^1^, which denotes that there was one canal in the buccal root in the area of the coronal orifice, which divided into two separate canals in the apical region. The third canal was located in the palatal root.

According to this new classification (Ahmed et al.) [15], in the Polish population, type ^2^FP B^1^P^1^ occurs most frequently, being found in more than half of cases, followed by type ^1^FP^2^, in 13% of the examined teeth. Hence, in the maxillary premolars of the Polish population, it is more common to detect two canals in two separate roots than in a single root. Similar results were obtained in a study of the anatomy of the maxillary premolars in an Egyptian subpopulation [10]. It should be noted that both the above-mentioned types/codes “make up” Vertucci type IV canal; however, they not only provide information about the presence of two canals, but also indicate the number of roots.

In the present study, ten other configuration types were also observed in the Polish population; however, as observed also by Saber et al. [16] (in an Egyptian subpopulation), there were significantly fewer of these than Vertucci type IV or code ^2^FP B^1^P^1^.

No significant difference in the prevalence of particular types of root canals was observed between men and women and between teeth 14 (right side) and 24 (left side). Similarly, Ng’ang’a et al. [8] did not find any significant differences in the distribution of particular types of canals between female and male teeth in Kenyans of African descent, based on the Vertucci classification. Similarly, Al-Nazhan et al*.* [42] did not identify significant differences in the distribution of the two root canals of the first maxillary premolar according to sex in a Saudi Arabian population. The procedure used by Ng’ang’a et al. [8] was based on decalcifying and clearing techniques in vitro, while Al-Nazhan et al. [42] employed radiological techniques.

Also, some authors have shown a difference in root canal system configuration in human permanent teeth between different age groups [43, 44]. Although the number of roots is not supposed to change over the years, since they are formed during the embryological stage of tooth development and are expected to remain similar in their outer morphology over time, the morphology of the root canal may indeed change.

In the case of roots, the changes occurring with age most often relate to the processes of resorption or layering of root cement/hypercementosis. However, this has no effect on root number. The situation is different for root canal morphology, especially in canals with an oval cross section and the presence of isthmus [43], as noted in a study of 12 325 teeth from 670 patients:

“Most of the root groups had higher or equal prevalence of Vertucci type I configurations in the younger groups whilst presenting a greater tendency for multiple root canal system configurations in older patients, mainly Vertucci type II in both maxillary and mandibular second premolars and in the distal root of the mandibular first molar” [43].

**Fig. 4:**
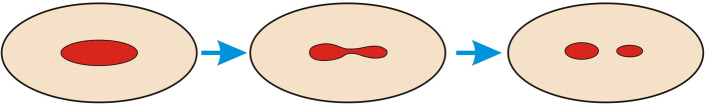
Diagram showing the change of the root canal system with age. In a young patient, there is one oval canal-type I according to the Vertucci classification. As a result of secondary dentition, in older patients, two canals are formed from a single canal—type II according to the Vertucci classification (if the canal is divided over its entire length)

Also, the first premolars of the maxilla, especially those with oval root canals and/or roots and severe obliteration processes, demonstrate a more complex canal system in older patients compared to younger patients [43]. It should be noted, however, that this predisposition to the presence of an additional canal (with age) is less noticeable in first maxillary premolars than in second maxillary premolars. The accumulation of secondary dentin with age leads to a reduction of the tooth chamber and a narrowing of the canals. In special cases, in the place of the isthmus and narrowing of the canal, there may be such a significant accumulation of secondary dentin that the canal will split into two separate canals at this point (Fig. 4). Very interesting observations on the formation of canals with age were presented by Peiris et al. [44] who note that in lower molars, the internal anatomy of the teeth is finally formed between the age of 30 and 40 years. In addition, the canals of young patients are wider and simpler in their structure than those in older patients.

In the present study, the mean age of the patients was about 40 years, so it can be assumed that the internal tooth anatomy was fully developed; this can also be assumed in the case of maxillary premolars, although additional analysis should be carried out using more accurate imaging of the root canal system.

Our study used cone-beam computed tomography (CBCT). This is the best method of radiological imaging in a clinical setting. The authors of many other studies on tooth anatomy have also used computed tomography to analyze the root canal system [3, 4, 20, 24, 26]. However, it should be noted that microtomography (microCT) is a much more accurate radiological method used to examine the morphology of the teeth. MicroCT scans are characterized by much higher resolution than CBCT images [45], allowing more precise examination and providing more detail [15, 16, 45, 46]. However, while our choice of CBCT may be regarded as a limitation of the present study, it is important to note that microCT cannot safely be used in patients in vivo due to its much higher radiation dose [16, 45].

However, a combination of volumetric imaging, careful observation of morphology on CBCT scans, and the use of accurate classification of roots and canals allows for better diagnostics in endodontics and other areas of dentistry [1, 2, 4].

## Conclusion

Maxillary first premolars display a wide range of root and canal anatomical variations. The new system for classifying canal morphology based on Ahmed et al. [15] is more accurate than the Vertucci classification. In vivo CBCT analysis is a noninvasive and clinically effective tool for examining root and canal morphology that may ultimately improve the outcome of endodontic treatment.

## Declarations

### Statistical analysis

Mrs. Irena Szymańska.
